# Cambogin Is Preferentially Cytotoxic to Cells Expressing PDGFR

**DOI:** 10.1371/journal.pone.0021370

**Published:** 2011-06-21

**Authors:** Ze Tian, Jie Shen, Fengfei Wang, Peigen Xiao, Junshan Yang, Hetian Lei, Andrius Kazlauskas, Isaac S. Kohane, Erxi Wu

**Affiliations:** 1 Institute of Medicinal Plant Development, Chinese Academy of Medical Sciences, Peking Union Medical College, Beijing, China; 2 Department of Pharmaceutical Sciences, North Dakota State University, Fargo, North Dakota, United States of America; 3 Department of Ophthalmology, Schepens Eye Research Institute, Harvard Medical School, Boston, Massachusetts, United States of America; 4 Informatics Program, Children's Hospital Boston, Harvard Medical School, Boston, Massachusetts, United States of America; Roswell Park Cancer Institute, United States of America

## Abstract

Platelet-derived growth factor receptors (PDGFRs) have been implicated in a wide array of human malignancies, including medulloblastoma (MB), the most common brain tumor of childhood. Although significant progress in MB biology and therapeutics has been achieved during the past decades, MB remains a horrible challenge to the physicians and researchers. Therefore, novel inhibitors targeting PDGFR signaling pathway may offer great promise for the treatment of MB. In the present study, we investigated the cytotoxicity and mechanisms of cambogin in Daoy MB cells. Our results show that cambogin triggers significant S phase cell cycle arrest and apoptosis via down regulation of cyclin A and E, and activation of caspases. More importantly, further mechanistic studies demonstrated that cambogin inhibits PDGFR signaling in Daoy and genetically defined mouse embryo fibroblast (MEF) cell lines. These results suggest that cambogin is preferentially cytotoxic to cells expressing PDGFR. Our findings may provide a novel approach by targeting PDGFR signaling against MB.

## Introduction

Medulloblastoma (MB) is the most common malignant central nervous system tumor in children, accounting for approximately 20% of all pediatric brain cancers [1], [2]. Despite the advances in understanding its biology, a cure is still elusive. Hence, there is an urgent need for developing new successful therapeutics in MB.

Multiple lines of evidence showed that platelet-derived growth factor alpha and beta receptors (PDGFRα and PDGFRβ) are co-expressed in MB and overexpressed in metastastic MB, which is highly associated with poor clinical outcome [2], [3]. In addition, the PDGFRs downstream mitogen-activated protein kinase (MAPK) signal transduction pathway is also upregulated in metastastic MB. Neutralizing antibodies to PDGFRα and MAPK specific inhibitor U0126 inhibited PDGFA-induced migration and blocked MAP2K1, MAP2K2 and MAPK1/3 phosphorylation in a dose-dependent manner [3], [4], [5]. Imatinib mesylate (Gleevec) is a successful PDGFR tyrosine kinase inhibitor for the treatment of some hematological malignant [6], dermatofibrosarcoma protuberans, and Kit^+^ Gastrointestinal Stromal Tumors (GIST) [7], [8], [9], [10]. Recent study revealed that imatinib induced apoptosis and inhibited cell proliferation as well as PDGF-BB- and serum-mediated migration and invasion in Daoy cells via blockade activation of PDGFRβ, Akt, and ERK [11]. These data suggest that inhibitors of PDGFRs should therefore be considered for investigation as possible novel therapeutic strategies against MB.

Natural products have been a wellspring of drugs and drug leads for decades and remain a major source for drug discovery. Some of the constituents from Garcinia species have demonstrated cytotoxic activity in different cancer cell lines [12], [13]. Our previous study has demonstrated that a xanthone derivate dulxanthone A induces cell cycle arrest and apoptosis via up-regulation of p53 through mitochondrial pathway in HepG2 cells [14]. Benzophenone derivatives and isoxanthochymol isolated from Garcinia genus showed significant growth inhibition and induction of apoptosis in human leukemia, breast cancer, colon cancer, and liver cancer cell lines as well [15]. Cambogin was isolated from *Garcinia cowa* and is an enantiomer of isoxanthochymol. Therefore, it is of great interest to examine the cytotoxic effect and mechanisms of cambogin in MB.

## Results

### Cambogin induces cytoxicity in MB and various solid tumor cell lines

**Figure 1:**
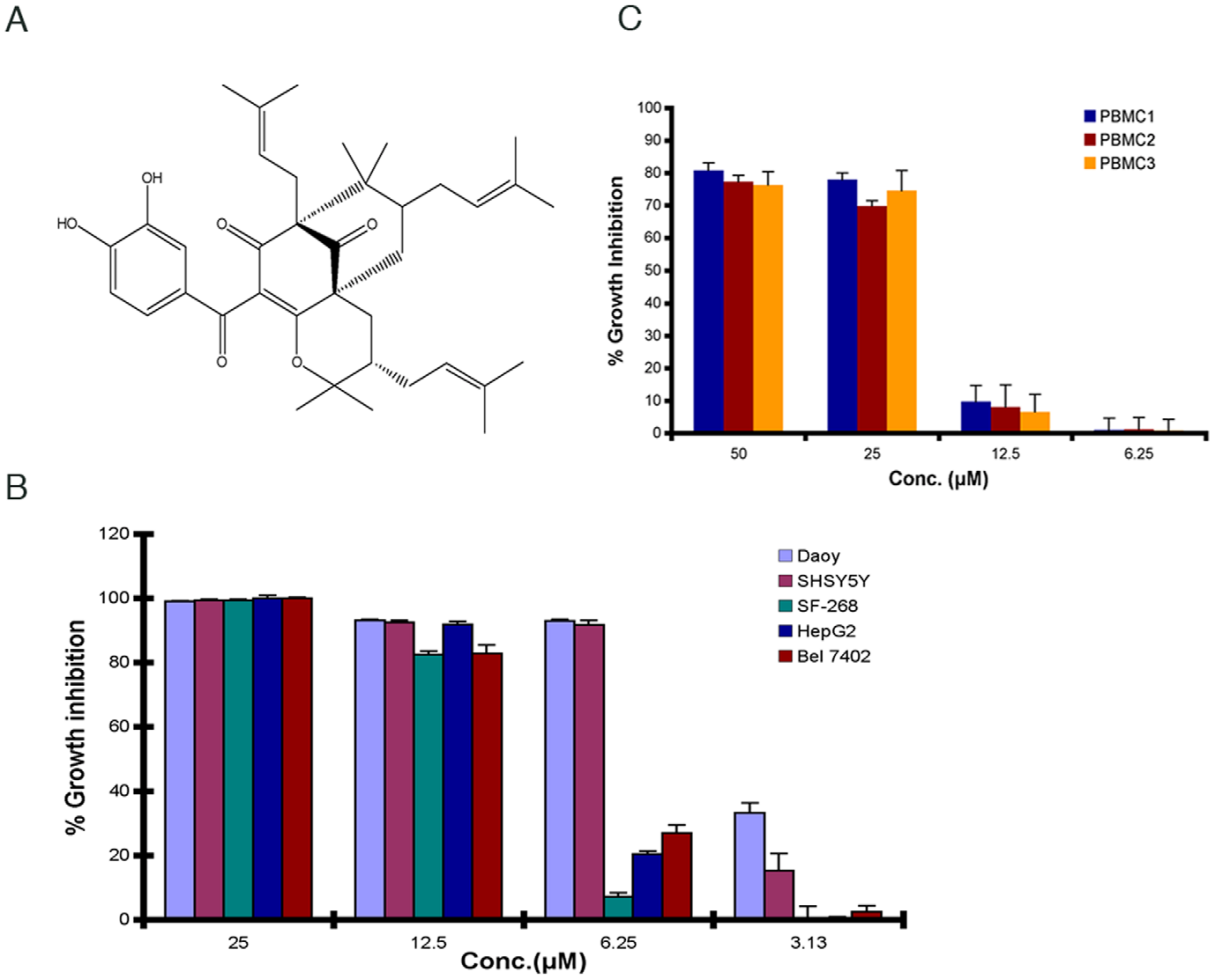
Cambogin triggers cell death in various cancer cell lines. A. Structure of cambogin; B. Cytotocicity of cambogin in various cancer cell lines Daoy (MB), SF-268 (glioblastoma), SHSY5Y (neuroblastoma), HepG2 (hepatoma), and Bel7402 (hepatoma) upon treatment for 48 h; and C. Cell viability of peripheral blood mononuclear cells (PBMC) upon treatment with cambogin for 48 h.

To determine the effect of cambogin in cancer cells, we first tested the cytotoxicity of cambogin in a panel of cancer cell lines using MTS assay. Our results show that following treatment with cambogin for 48 h at indicated concentrations, significant of cytotoxicity was observed in all tested cell lines Daoy (MB), SF-268 (glioblastoma), SHSY5Y (neuroblastoma), HepG2 (hepatoma), and Bel7402 (hepatoma) in a dose-dependent manner (Fig 1B). Among them, MB cell line Daoy is most sensitive to cambogin treatment. Importantly, cambogin did not affect the cell viability of PBMCs from health donors at the comparable dosage (Fig 1C).

### Cambogin blocks cell cycle at S phase in Daoy cells

**Figure 2:**
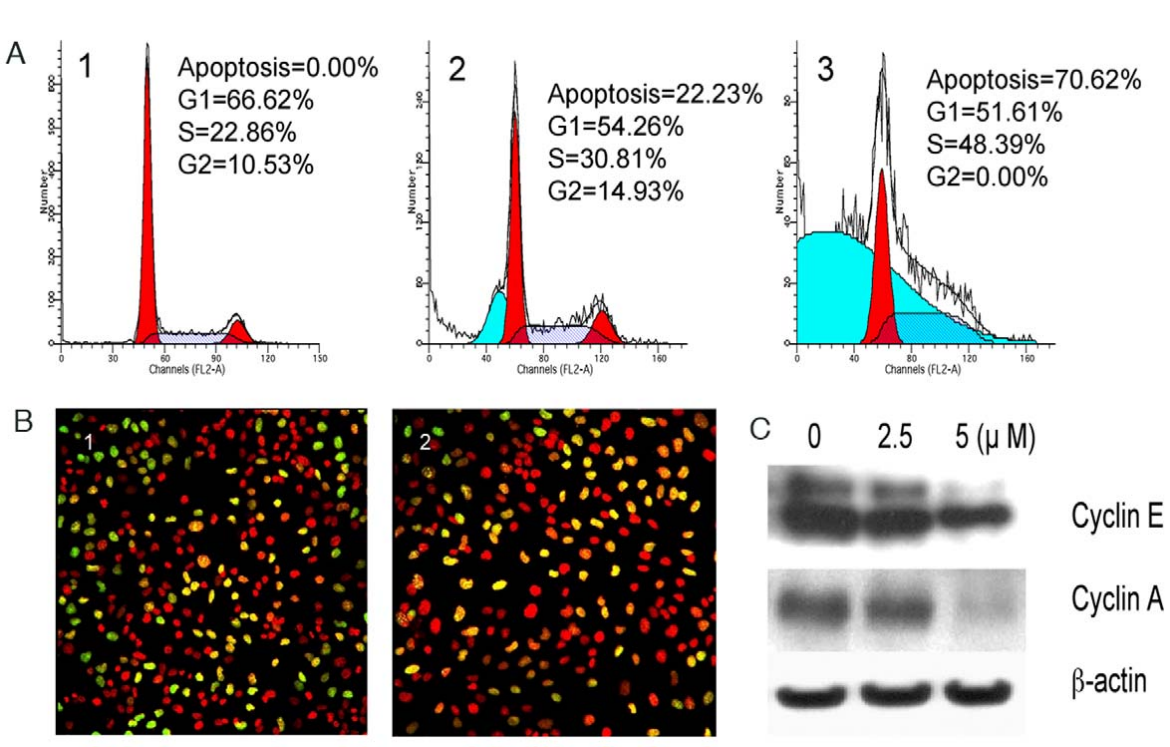
Cambogin triggers S phase arrest in Daoy cells via down regulation of cyclin A and E. A. Cambogin induces S phase cell cycle arrest in Daoy cell line. Daoy cells were treated with cambogin for 48 h and followed by flow cytometry analysis. 1, control cells; 2-3, treatment with 2.5 and 5 µM cambogin, respectively. B. Cambogin inhibits DNA synthesis in Daoy cells using BrdU Incorporation Assay. 1, control Daoy cells; 2, Daoy cells treatment with cambogin at 5 µM for 24 h. C. Cambogin downregulates cyclin A and E using Western blotting. β-actin was used as internal loading control.

Many anti-tumor agents act at multiple steps in the cell cycle. The ability of a compound to affect specific phases of the cell cycle could provide a clue to its cytostatic or cytotoxic mechanism of action. Following treatment of Daoy cells with cambogin at different concentrations, a persistent accumulation of S phase and apoptotic cells was observed (Fig 2A). In addition, treatment with cambogin at 5 µM for 24 h dramatically inhibited DNA synthesis, which was confirmed by BrdU incorporation assay. The green signal from BrdU was less than that in the control (Fig 2B). Consistent with cell cycle arrest at S phase, cyclin A, and cyclin E were down regulated by cambogin (Fig 2C).

### Cambogin triggers apoptosis in Daoy cells

**Figure 3:**
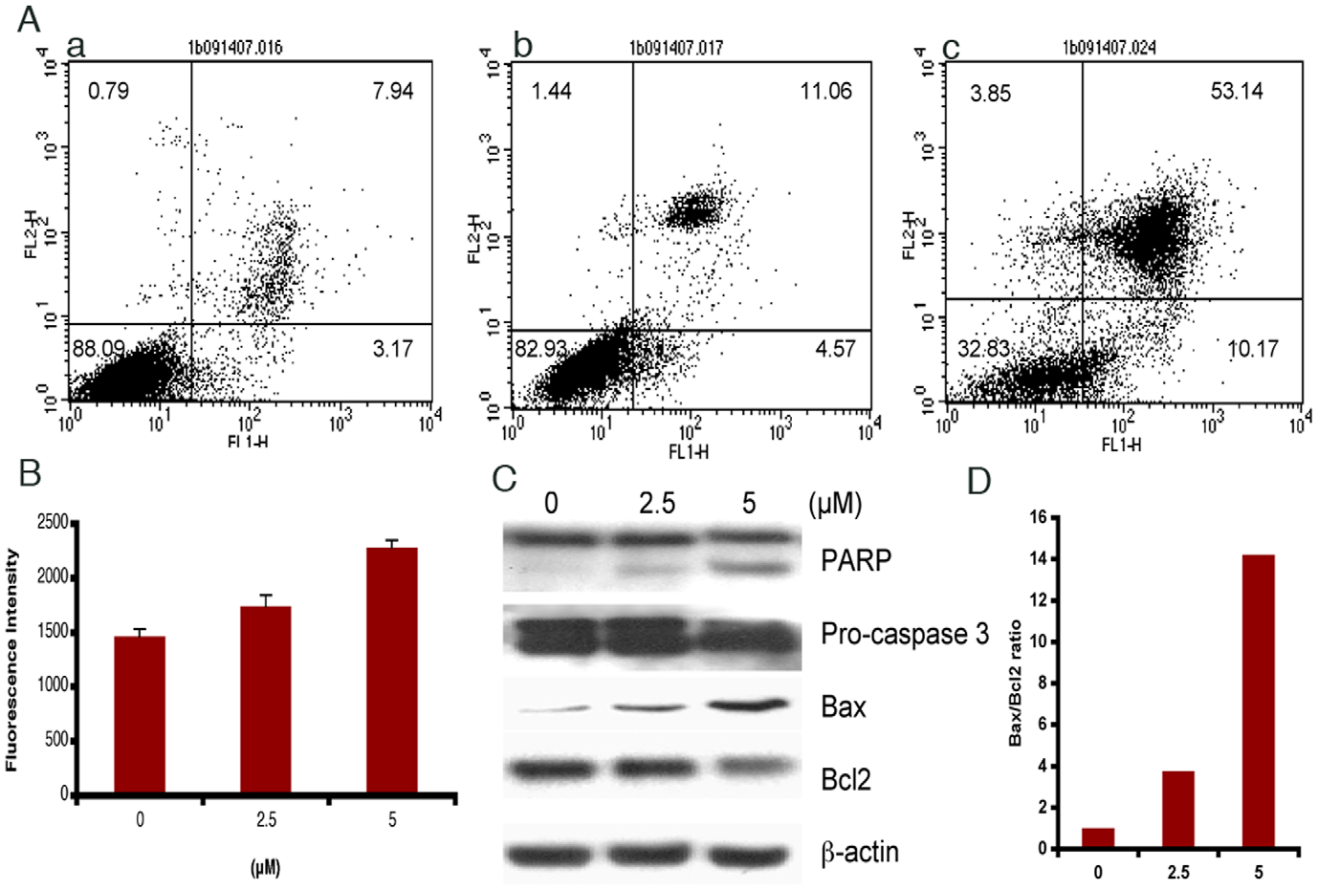
Cambogin triggers apoptosis in Daoy cells. A. Cambogin induces apoptois in Daoy cells. a-c, Daoy cells treatment with cambogin at 0, 2.5, and 5 µM for 24 h, and then apoptosis was quantified by Annexin V/PI staining. B. Treatment with cambogin at 2.5 and 5 µM for 48 h activates caspase 9. C. Treatment with cambogin at 2.5 and 5 µM for 48 h altered apoptotic regulatory protein expression. D. Protein expression ratio of Bax/Bcl2 was calculated by NIH ImageJ with correction using β-actin.

To determine whether the reduction in viability of cancer cells by cambogin occurred via induction of apoptosis, we used Annexin V-FITC/PI double staining to quantify apoptosis in treated Daoy cells. After treatment with cambogin at 2.5 and 5 µM for 24 h, we observed significant induction of apoptosis evidenced by the increase of Annexin-V^+^PI^-^(early apoptosis) and Annexin-V^+^/PI^+^(late apoptosis and necrosis) populations (Fig 3A).

The induction of apoptosis may also involve activation of caspases. In the light of our study, cambogin induced caspase 9 activity and reduced downstream procaspase 3 protein expression. Furthermore, treatment with cambogin also induced PARP cleavage and bax/bcl2 ratio (Figs 3B-D).

### Cambogin inhibits PDGFRα & β pathway

**Figure 4:**
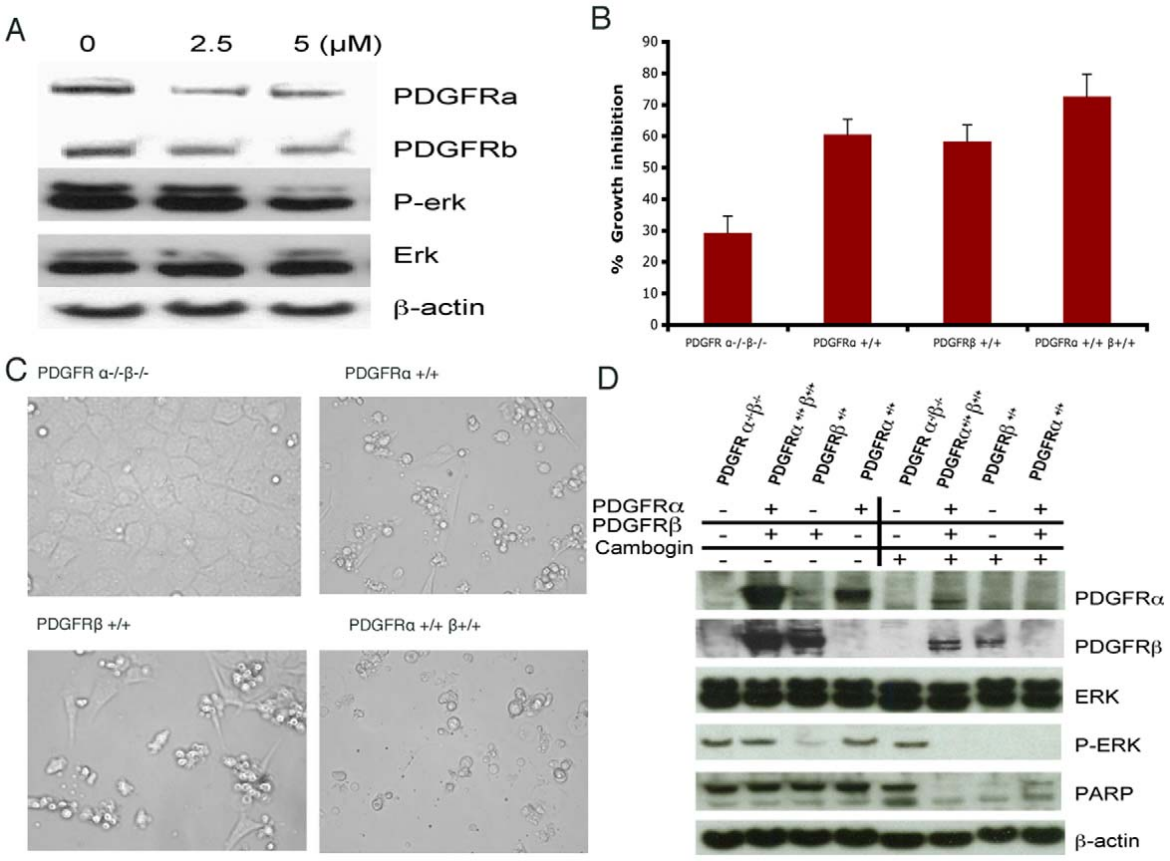
Cambogin triggers cell death via preferentially PDGFRs pathway. A. Cambogin down regulated PDGFR signaling in Daoy cells using Western blotting. B and C. Cambogin induced significant cell death in MEF cell lines with PDGFR signaling compared with PDGFR signaling null cells. The cell growth was measured by MTT and photograph was taken under the microscope. D. Cambogin down regulates PDGFR signaling (PDGFRα, PDGFRβ, erk, p-erk, and PARP) in genetically defined MEF cell lines using Western blotting (β-actin was used as internal loading control). Four cell lines (PDGFRα ^+/+^; PDGFRβ ^+/+^, PDGFRα ^+/+^ β^+/+^ and PDGFR α^−/−^β^−/−^ cells) were used as indicated.

PDGFR signaling has been implicated in the pathogenesis of variety of cancer types and therefore it is a rational target for anticancer agents [16]. We examined the effect of cambogin on PDGFR signaling pathway. Our results show that cambogin decreased the expression of PDGFRα and β and the downstream p-erk expression in Daoy cells (Fig 4A). To confirm the data in Daoy cells, we further investigate the influence of cambogin on PDGFR signaling using a panel of genetically defined MEF cell lines. Following the treatment of MEF cell line with cambogin at 5 µM for 24 h, significant cell death was observed in PDGFRα ^+/+^, PDGFRβ ^+/+^, and PDGFR^α +/+ β+/+^ cells. However cambogin did not markedly affect the viability of PDGFR α^−/−^β^−/−^ cells (Figs 4B and C). Furthermore, in agreement with the observation in Daoy cells, cambogin dramatically suppressed the protein expression of PDGFRα & β and p-erk, triggered the cleavage of PARP in PDGFRα ^+/+^, PDGFRβ ^+/+^, and PDGFRα ^+/+^ β^+/+^ cells in comparison with PDGFR α^−/−^β^−/−^ cells.

## Materials and Methods

### Cell Culture and Drug Treatment

Daoy, SF-268, SHSY5Y, HepG2, and Bel7402 cells (obtained from ATCC, Manassas, VA) were cultured in RPMI 1640 complete medium supplemented with10% FBS (fetal bovine serum), 100 units/ml penicillin, 100 µg/ml streptomycin, and 2 mM L-glutamine at 37°C and 5% CO2. Genetically defined MEF cells (PDGFRα ^+/+^; PDGFRβ ^+/+^, PDGFRα ^+/+^ β^+/+^ and PDGFR α^−/−^β^−/−^ cells) were cultured in DMEM complete medium [17]. Cells were grown to 70% confluence, trypsinized with 0.25% trypsin, 2 mM EDTA, and seeded 24 h prior to treatment in all experiments. Peripheral blood mononuclear cells (PBMCs) were isolated from normal healthy donors by Ficoll and cultured with RPMI 1640 complete medium [18].

Cambogin was isolated from stems of *Garcino cowa* and identified by comparing the EI-MS, 1H, and 13C NMR data with the literature [19] and dissolved in DMSO at a concentration of 100 mM then diluted in tissue culture medium and filtered before use. The concentration of DMSO (0.1%) did not affect the cell viability.

### Cytotoxicity Assay

Cytotoxicity of cambogin in cell line and normal PBMCs was assessed by MTS (3-(4,5-dimethylthiazol-2-yl)-2,5- diphenyltetrazolium bromide; Chemicon International Inc, Temecula, CA) and CellTiter-Glo (Progema, Madison, WI) assays according to the manufacturer's instructions.

### Apoptosis and Cell Cycle Analysis

Daoy cells were treated with 2.5 and 5 µM cambogin at indicated times and followed by flow cytometry analysis. Apoptosis was quantified using Annexin V-FITC/ propidium iodide detection kit (BD Biosciences, Clontech, CA) and the data were analyzed with FACSCalibur (BD Bio- sciences). Cell cycle distribution was determined by PI staining and the percentage of cells in different cell cycle phases was calculated by ModFit 3.0 (Verity Software House) [14].

### Western Blotting

Following treatment with cambogin at 2.5 and 5 µM for 24 h, Daoy cells were harvested and lysed in RIPA buffer. Equal amounts of proteins were subjected to NuPAGE 4-12% Bis-Tris Gel (Invitrogen, Carlsbad, CA) for electrophoresis and then transferred onto nitrocellulose membranes (Amersham, Piscataway, NJ). The membrane was then incubated with anti-PDGFRα, anti-PDGFRβ, anti-cyclin A, anti-cyclin E, anti-caspase 3, anti-PARP, anti-Bax, anti-Bcl2, anti-perk, anti-erk and, anti-β-actin antibodies (Santa Cruz Biotechnology, Santa Cruz, CA). Secondary antibody to IgG conjugated to horseradish peroxidase was used (Bio-Rad, Hercules, CA). Protein bands were visualized with the ECL Western blotting detection system (Thermo Scientific, Rockford, IL) according to the manufacturer's instructions.

### Caspase Activity Assay

Caspase-9 detection kit (QIA 115, Calbiochem, Darmstadt, Germany) was used to measure caspase-9 activity in live cells using fluorescent detection. Daoy cells were seeded in 6-well plates. Following treatment with vehicle or cambogin at indicated concentrations for 48 h, cells were harvested and resuspended with 300 µl medium. About 1 µl of FITC-LEHD-FMK was added to each sample (one as blank) and incubated at 37°C, 5% CO2 for 0.5 h. The samples were centrifuged, washed and resuspended in 100 µl wash buffer. Cells were then transferred to a 96-well fluorescent plate and the fluorescence intensity was measured at Ex. 485 nm and Em. 535 nm. One-factor ANOVA was used and p<0.05 was considered as significant.

### BrdU Incorporation Assay

Daoy cells were treated with vehicle or cambogin at 5 µM for 24 h, and then cells were incubated with 20 mM bromodeoxyuridine (5-bromo-2-deoxyuridine, BrdU) for another 1 h before fixation with acetone (-20°C) for 10 min. After DNA denaturation in 2 N HCl at 37°C for 30 min, Daoy cells were blocked with PBSST for 30 min, followed by incubation with BrdU antibody (10 µl BrdU-Alexa 488/647 with DAPI 1 µl into 90 µl PBSST) for 1 h at room temperature [20]. Pictures were taken under fluorescence microscopy.

## Discussion

Natural products have made, and continue to make, an indispensable contribution to the discovery and development of effective drugs for the treatment of human malignancies. In the current study, we first investigated the cytotoxicity and the mode of action of cambogin in MB Daoy cells.

Our results show that combogin triggers significant cell death in various cancer cell lines and exhibits higher sensitivity in MB Daoy cells. By contrast, cambogin did not markedly affect the cell viability of normal PBMCs. This indicates the specific anti-tumor activity and a favorable therapeutic index of combogin. In addition, induction of the S phase cell cycle arrest and apoptosis have been implicated as an important mechanism underlying the cytotoxic activities of cambogin-induced Daoy cell death. Following the treatment of cambogin, Daoy cell cycle was arrested at S phase and DNA synthesis was prominently inhibited. At the same time, cyclin A and E, the cyclins that govern cell cycle progression were down regulated. Furthermore, activation of caspases and increase of bax/bcl2 ratio contribute to cambogin-triggered apoptosis in Daoy cells as well.

PDGF receptors and/or ligands are overexpressed in a variety of cancers [16], [21]. In the past decade, a number of inhibitors of receptor tyrosine kinases (RTKs) whose targets include PDGFR have emerged as therapeutics with remarkable efficacy in the treatment of human malignancies [22], [23], [24], [25], [26].Two reported studies have shown that PDGFRα-neutralizing antibodies and siRNA silencing of PDGFRβ, respectively, inhibited signaling, migration, and survival in MB Daoy cells [3], [11], indicating that PDGFRs may be therapeutic targets for MB. Hence, a novel structure targeting PDGFR pathway might offer great promise as therapeutics in MB. In this context, it is of particular interest to examine the influence of cambogin on PDGFR signaling pathway. Our further mechanistic study on cambogin-induced apoptosis revealed that cambogin inhibits PDGFR signaling by down-regulation of the protein expression of both PDGFRα and β, as well as their downstream p-erk in Daoy cells. To confirm our data in MB, we utilized PDGFRα^+/+^, PDGFRβ^+/+^, PDGFRα^+/+^ β^+/+^, and PDGFR α^−/−^β^−/−^, a panel of genetically defined MEF cell lines to examine the influence of cambogin on PDGFR signaling. Similar to our findings in MB cells, cambogin showed pronounced killing in PDGFRα^+/+^, PDGFRβ^+/+^, and PDGFRα^+/+^ β^+/+^ MEF cells, which have either PDGFRα or PDGFRβ signaling transduction, but little killing in PDGFRα^−/−^β^−/−^ cells lacking PDGFR signaling. This indicates that PDGFR containing cells were highly sensitive to cambogin. Viability of the PDGFR α^−/−^β^−/−^ cells lacking PDGFRs may be explained by the phenomenon of “oncogene addiction” [27]. In addition, cambogin down-regulated either PDGFR α and/or β and p-erk in PDGFRα^+/+^, PDGFRβ^+/+^, and PDGFRα^+/+^ β^+/+^cells. However, p-erk did show dramatic reduction in PDGFR null cells. Furthermore, cambogin induced more apoptosis in MEF cell lines with PDGFR signaling evidenced by dramatically decrease in full length PARP compared with PDGFR null cells. Overall, these data suggest that down regulation of PDGFRα and /or PDGFRβ could at least partially explain cambogin-induced apoptosis.

In conclusion, our results demonstrate that cambogin is preferentially cytotoxic to cells expressing PDGFR. Novel inhibitors targeting PDGFR signaling pathway could provide an effective strategy for the treatment of MB.
